# Hidden state prediction: a modification of classic ancestral state reconstruction algorithms helps unravel complex symbioses

**DOI:** 10.3389/fmicb.2014.00431

**Published:** 2014-08-25

**Authors:** Jesse R. R. Zaneveld, Rebecca L. V. Thurber

**Affiliations:** Vega Thurber Laboratory, Department of Microbiology, Oregon State UniversityCorvallis, OR, USA

**Keywords:** predictive metagenomics, “virtual” metagenomes, 16S rRNA gene copy number, phylogenetic prediction, systems biology, ecotoxicology

## Abstract

Complex symbioses between animal or plant hosts and their associated microbiotas can involve thousands of species and millions of genes. Because of the number of interacting partners, it is often impractical to study all organisms or genes in these host-microbe symbioses individually. Yet new phylogenetic predictive methods can use the wealth of accumulated data on diverse model organisms to make inferences into the properties of less well-studied species and gene families. Predictive functional profiling methods use evolutionary models based on the properties of studied relatives to put bounds on the likely characteristics of an organism or gene that has not yet been studied in detail. These techniques have been applied to predict diverse features of host-associated microbial communities ranging from the enzymatic function of uncharacterized genes to the gene content of uncultured microorganisms. We consider these phylogenetically informed predictive techniques from disparate fields as examples of a general class of algorithms for Hidden State Prediction (HSP), and argue that HSP methods have broad value in predicting organismal traits in a variety of contexts, including the study of complex host-microbe symbioses.

## BIOLOGICAL DIVERSITY OFTEN NECESSITATES TRAIT PREDICTION

The immense scope of biological diversity limits detailed scientific study to a relatively small number of well-characterized model organisms. Because the technical and analytical capabilities needed to catalog the vast number of diverse organisms are limited, important scientific and regulatory decisions must often be made by applying information from well-studied models onto less well-understood organisms (Fagan et al., 2013; Guénard et al., 2013) or genes (Eisen, 1998; Engelhardt et al., 2005).

In some cases, extrapolation of properties across diverse organisms is needed because direct testing on the organism of interest would be unethical, illegal, and/or infeasible. For example, in the realm of ecotoxicology, direct toxicology tests on suitably large cohorts of endangered or threatened species (e.g., spotted owls or marine mammals) may better predict the lethal concentration of a toxicant than tests on other model species, but would be legally problematic and potentially counterproductive from the standpoint of conservation. Instead, data from experiments on model species must generally be extrapolated to predict impacts in rare or hard to access relatives (Guénard et al., 2011).

These problems of vast diversity and limited ability to study all species are particularly apparent in the context of complex symbiotic assemblages like those between metazoans and their associated microbial communities. The human gut lumen has been estimated to contain ~1000 prevalent microbial species and ~3.3 million genes based on surveys of European populations (Qin et al., 2010). The total microbial cell count on humans (~10^14^ cells) is even estimated to exceed that of human cells (~10^13^) by roughly an order of magnitude (Savage, 1977). The connections between the human microbiota and a wide range of variables including diet, autoimmune disease, obesity, and cancer are being actively explored (see Lozupone et al., 2012 for a recent review). Although the human microbiome is a heavily studied system, the diversity of its constituents presents an important challenge to gaining an ecosystem-level understanding of the contribution of each member to the dynamics of the overall system.

This immensity of microbial diversity presents an even larger challenge when considering less well-studied microbial communities. The Greengenes database (McDonald et al., 2012) of microbial 16S ribosomal RNA gene sequences, a popular phylogenetic marker for bacteria and archaea, contains 99,322 microbial operational taxonomic units (OTUs) at a 97% sequence similarity threshold in the present version (13_8). Even if representatives of 100 OTUs per day could be cultured and assayed for a particular trait (an effort that would require extensive resources and automated methods for high-throughput phenotyping), it would take ~6 years to test this trait across known OTUs. Unfortunately, this is slower than discovery of new OTUs, which tripled between 2012 and 2013. Thus even an impressive brute-force effort to study microbial phenotypes by treating each OTU in isolation would actually lose ground to the influx of newly uncovered microbial diversity. Therefore, many conclusions about microorganisms found in the environment will rely on the properties of better-studied model microorganisms for some time, especially if new microbial diversity continues to be uncovered at high rates.

## METHODS FOR PHYLOGENETIC PREDICTION

In this review, we discuss the utility of phylogenetic models for predicting features of understudied organisms, and focus in particular on a group of methods for predicting unknown traits from a phylogeny that we term Hidden State Prediction (HSP) algorithms. These methods have recently been applied to a variety of interesting problems in the study of host-microbe symbioses and microbial ecology.

**FIGURE 1:**
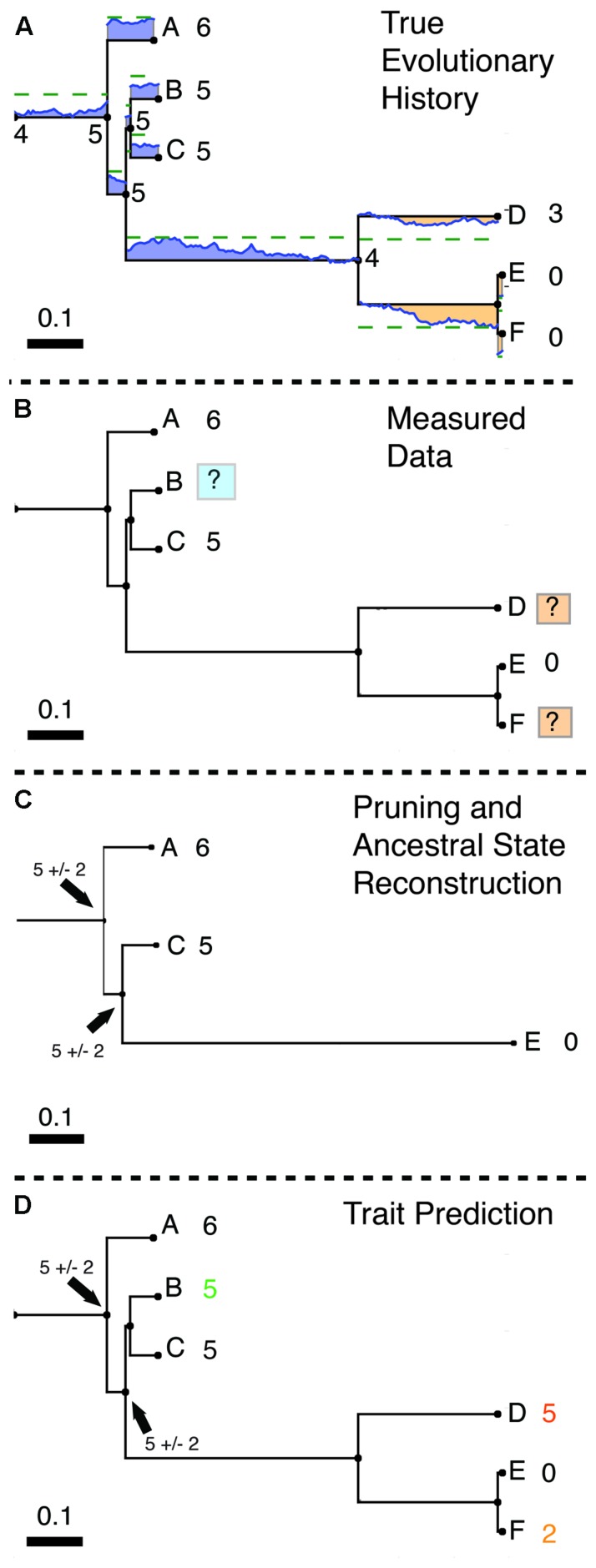
**Hidden State Prediction (HSP). (A)** Evolution of a simulated trait following a Brownian motion model. For example, the copy number of a gene family in each of several microbial genomes can be mapped onto a phylogenetic tree and represented as a continuous trait. (The same method could be used on any continuous evolutionary character.) Here, a trait starting with a value of 4 evolves by a Brownian motion process within a group of organisms A–F. Blue values above each edge of the phylogeny indicate regions of the phylogeny where the trait takes on a value greater than 4 (gain with respect to the ancestor of A–F). Orange values below the edges indicate trait values lower than 4 (loss relative to the ancestor). Numbers by the tips of the tree show the final value of the trait rounded to the nearest integer, as when the trait is taken to represent the copy number for a particular gene. (**B)** Observed Data. In general only a portion of all modern organisms are sampled. In this example trait values have been measured for tips A, C, and F but are unknown for tips B, D, and E.The tips with unknown trait values differ in their proximity to characterized relatives. Tip D is only distantly related to tips with known values. Note that tip B is closely related to tips A and C for which trait values are known. Thus for B the closest known tip is within 0.12 units of branch length, whereas for D the closest tip is 0.63 units of branch length away. The task of HSP is to estimate trait values for B, D, and F from the values for A, C, and E. Examples of tips for which trait prediction will be more or less accurate are shaded with blue or orange boxes, respectively. This task will be simplest in cases like B in which several close relatives have been assayed and hardest in cases like D where long branches separate unknown tips from known references. **(C)** Ancestral State Reconstruction (ASR). The unknown tips are dropped from the tree (most phylogeny programs cannot handle missing character values) and ancestral character values are calculated for the remaining internal nodes. (An alternative method for discussed in the text is to repeatedly reroot the tree at each node of interest (here B,D,F) and perform standard ASR (Garland and Ives, 2000). (**D)** Prediction of character values. If prediction via tree rerooting is not used, the inferred ancestral states and evolutionary model must be extended to the tips using another method. For example, the predictive functional profiling software package PICRUSt (which predicts metagenomic counts from marker gene data; see main text) uses exponential weighting by branch length to extend reconstructed states to the tips, and inflates the variance of the reconstructed ancestral state to account for evolution between the ancestor and the tip of interest (Langille et al., 2013). In this example, Tip B, with close references A and C is assigned correctly. Tips D and F, where such references are either missing (tip D) or available only in a sister group (but not a closely related outgroup; F) are assigned less accurately (both off by two copies). However, D is correctly inferred to have more copies than F. Note that this example is intended to illustrate compactly the algorithm and some examples of success or failure, and should not be taken to represent the average accuracy of these methods, which have been studied in some depth (see Factors Influencing the Accuracy of Hidden State Prediction Algorithms for a summary of major findings).

We define HSP algorithms as phylogenetic methods for predicting unknown character states or character values (i.e., traits) based on a collection of known character states and a phylogenetic tree (**Figure 1**). Thus, HSP is similar to ancestral state reconstruction (ASR) techniques, in which the properties of ancestral organisms are inferred based on traits of their living descendants. However, HSP methods differ from ASR methods in that the properties of modern rather than ancestral organisms are predicted. That is, these methods predict character states for the tips of a phylogeny rather than for its internal nodes. These methods are also closely related to phylogenetic comparative methods (PCMs), which also examine the predictability of character values given a phylogeny, but do so in order to *remove* phylogenetic signal when comparing traits of interest (Harvey and Pagel, 1991; Garland and Ives, 2000). Although the two methods are closely related, we use HSP to distinguish phylogenetic prediction *per se* from methods where a phylogenetically corrected comparison of *measured* trait values is the end goal. Finally, we should be clear that while HSP methods are also sometimes called “phylogenetic predictive methods,” they are distinct form standard phylogenetic inference: HSP methods use an inferred phylogeny to predict traits at the tips of the tree rather than *vice versa.*

### HIDDEN STATE PREDICTION ALGORITHMS

Consider the case of predicting the copy number of a gene across many organisms, of which only a portion have been characterized (**Figures 1A,B**). HSP methods start with a set of reference annotations (the gene copy numbers) and a phylogenetic tree relating the entities that were annotated (here the organisms carrying the genes). These reference annotations are mapped onto the corresponding tips of the tree. (Although this step is conceptually simple, it can actually be surprisingly involved when reference databases and the reference phylogeny use different conventions and naming schemes.)

When the model of evolution is reversible over time (e.g., in a Brownian motion model), it is possible to make phylogenetic predictions for hidden states directly using ASR of a rerooted version of the phylogeny (Garland and Ives, 2000). Because the direction in which time moves is not important in such models, the problem of prediction can be transformed into a standard ASR by rerooting the tree on the parent edge for the node to be predicted, and resolved using any standard ASR method. Fast reconstructions using phylogenetic independent contrasts or generalized least squares (which have been shown to be equivalent for the Brownian motion model specifically) are a popular choice (Grafen, 1989; Martins and Hansen, 1997; Felsenstein, 2004). However, due to technical limitations of most phylogenetic software, it may be necessary to prune all tip nodes with unknown character values prior to prediction. The combined rerooting and pruning operations may incur significant computational costs on extremely large reference phylogenies common in microbial studies (e.g., 10s of 1000s of tips). Nonetheless, this method has been used by several recent phylogenetic prediction studies on large microbial trees (Kembel et al., 2012; Angly et al., 2014).

Alternatively, a standard ASR can be performed on the pruned tree (**Figure 1C**), then mapped back to the full tree, and the inferred ancestral states and evolutionary model used to predict character values at nodes removed during pruning and the tips of the tree (**Figure 1D**). Under maximum parsimony (Fitch, 1971), which seeks to minimize character state changes over the tree, the most parsimonious hidden state is simply the trait value reconstructed for the last common ancestor of a tip and its closest annotated relative. As an example of this approach, (Eisen, 1998) suggested the use of a parsimony-based HSP algorithm to predict the gene function of unassigned orthologs within gene families. In a maximum likelihood framework the most likely prediction for the hidden state is the one that maximizes the likelihood of the observed character data given the phylogeny and a particular model of evolution (alternative models may be tested using Akaike information criterion/Bayesian information criterion approaches). For symmetrical models of character evolution (such as the Brownian motion model), this criterion implies that the ML estimate of a hidden state at the tip of the tree will be the same as the ML estimate of the ancestral state for the last common ancestor of the individual in question and an annotated relative. The variance in this trait will be inflated by the product of the variance parameter describing the Brownian motion process (σ^2^) and the branch length to account for evolution along the branch from the last common ancestor to the tip in question. If the model of evolution is asymmetrical, then the maximum likelihood estimate for a tip may differ from its last common ancestor with an annotated tip.

Because HSP methods predict features of modern organisms, the accuracy of these algorithms can be readily tested by cross-validation. When a large number of directly observed character values are known, the accuracy of an HSP method can be assessed by limiting program input to a subset of observed character values, and then testing the ability of the method to predict the rest. (The key conclusions from several such cross-validation studies are discussed in section “Factors Influencing the Accuracy of Hidden State Prediction Algorithms,” below.)

#### Related prediction methods

Several related approaches bear mentioning that also aim to extend information from characterized organisms to uncharacterized relatives. Phylogenetic eigenvector maps (PEMs) translate a phylogenetic tree into a matrix of similarities, and then decompose these similarities into orthogonal eigenvectors (Guénard et al., 2013). Some or all of these eigenvectors are then used as predictor variables in statistical analysis. This approach is similar to performing principal coordinates analysis (PCoA) on the similarity matrix of organisms, and then using some or all of the resulting PC axes as variables for statistical analysis. This method is implemented in the MPSEM R package, and has been used to predict the sensitivity of diverse animals to environmental toxicants (Guénard et al., 2011). Other approaches average gene counts across taxa or close phylogenetic relatives to estimate trait values. For example, Okuda et al. (2012) used all neighboring taxa within an empirically defined phylogenetic distance (0.10 16S rRNA subst./site was recommended) to predict metagenome contents from DGGE bands.

Finally, taxonomic binning approaches do not use a phylogenetic tree, but instead average values within taxonomic units in order to estimate the chances that uncharacterized taxa share that trait. For example, PanFP^1^ (Jun et al., Unpublished) seeks to normalize 16S rRNA copy numbers and predict microbial metagenomes from 16S rRNA data using this method. Careful comparison of the performance of HSP and each of these alternative methods in a variety of scenarios will be a valuable tool in guiding the development of methods for trait prediction.

## APPLICATION OF HIDDEN STATE PREDICTION TO UNDERSTAND COMPLEX SYMBIOSES

In the remainder of the paper we will discuss applications of HSP in the study of microbial symbioses, which range from correcting long-understood biases in 16S rRNA surveys to approximate predictions of the content of microbial genomes and metagenomes from amplicon data.

By allowing more accurate estimation of the composition of microbial communities (see Quality Control of Marker Gene Surveys through Copy Number Normalization), and their functional capabilities (see Phylogenetic Prediction of Microbial Genomes and Metagenomes), HSP methods are being used to study the interactions of complex communities of microrganisms with hosts and one another.

### QUALITY CONTROL OF MARKER GENE SURVEYS THROUGH COPY NUMBER NORMALIZATION

Recently HSP methods have been used to address a long-standing problem in microbial ecology. Enormous progress has been made in exploring complex microbial communities through sequencing of phylogenetically informative marker genes. The 16S rRNA is the most widely used marker gene for studies of bacteria and archaea. However, bacteria and archaea vary in 16S rRNA gene copy number from a mode of 1 copy (Angly et al., 2014) to 15 copies in *Photobacterium profundum*. Therefore the relative abundance for certain species and broader taxa inferred from qPCR or 16S rRNA sequencing can be inflated. Bias due to 16S rRNA gene copy number is expected to affect some datasets more than others, depending on the magnitude of differences in 16S rRNA genomic copy numbers for the most abundant organisms.

Recently, several publications have described the use of HSP to correct bias due to variation in 16S rRNA copy number in microbial datasets. Kembel et al. (2012) introduced a method for predicting 16S rRNA copy numbers for uncultured microorganisms, and used the prediction to normalize 16S rRNA marker gene surveys. This method inserts reference taxa into a phylogenetic tree for a particular community, and then uses HSP via rerooting and ASR to estimate missing 16S rRNA copy numbers.

PICRUSt^2^, a newly developed program for prediction of microbial genomes and metagenomes from 16S rRNA data (discussed below), also corrects 16S copy number using HSP and either PIC, ML, or parsimony reconstructions (Langille et al., 2013), but precalcuates results on the Greengenes tree rather than using tree-insertion on user datasets.

CopyRighter (Angly et al., 2014) is a third method for estimating and correcting 16S rRNA copy number which, like Kembel et al. (2012) uses the method of phylogenetic contrasts and rerooting to predict hidden states (Garland and Ives, 2000), but, like PICRUSt, pre-calculates predictions for each OTU.

These and related methods will likely prove to be a common quality-control step in 16S rRNA-based microbial ecology pipelines.

### PHYLOGENETIC PREDICTION OF MICROBIAL GENOMES AND METAGENOMES

The HSP methods used to predict 16S rRNA gene copy number for normalization purposes have also been extended across all genes in bacterial genomes to predict the genome contents of uncultured bacteria and archaea.

By combining gene predictions for each OTU with 16S rRNA copy number normalization it is possible to estimate the metagenome contents of entire microbial communities. This is useful because although metagenomic data can be collected directly using shotgun sequencing, this is presently quite expensive relative to surveys of a particular amplicon. (Metagenomic sequencing is so expensive relative to amplicon sequencing in part because sequencing depth must be sufficient to cover both genes and taxa, rather than just taxa.)

The PICRUSt software package uses HSP to estimate the copy number of each gene family across all OTUs in a reference phylogeny (by default the reference Greengenes 16S rRNA phylogeny). PICRUSt can use several different ASR methods at the user’s discretion including Wagner parsimony, Maximum Likelihood or phylogenetic independent contrasts. 16S rRNA copy numbers for each OTU are also estimated using HSP.

The product of these initial steps is a table of predicted gene and 16S rRNA copy numbers for each microorganism in the reference tree, including the many OTUs for which no genome sequence data is available. 95% confidence intervals for these gene copy numbers can also be constructed, based on the model of evolution for each gene. The resulting estimates of gene family and 16S rRNA copy number in each of the OTUs on the Greengenes tree can then be combined to predict “virtual” metagenomes from 16S rRNA data.

To do so, the observed count of 16S rRNA sequences in each OTU from a 16S rRNA amplicon library is simply divided by the predicted 16S copy number. As described above, this step produces an estimate for the relative abundance of each microbial OTU. The normalized counts for each OTU are then multiplied by the vector of gene abundances to produce an estimate of the count of each gene family in the metagenome.

Estimation of metagenome contents using HSP has already been applied to several studies of host-microbial symbiosis. McHardy et al. (2013) used PICRUSt to compare microbial diversity, predicted gene function, and observed metabolomic profiles in the human gut, and found PICRUSt predictions largely concordant with bulk metabolite profiles obtained by mass-spectroscopy. Rooks et al. (2014) applied PICRUSt to study gene families enriched in the gut microbiome in colitis, and found significant enrichment of several gene families previously implicated in the literature. Davenport et al. (2014) examined the role of diet on the gut microbiota in Hutterite populations. Summer diets containing more fruits and vegetables corresponded to higher levels of genes in the Glycan biosynthesis and degradation KEGG category. This increase was attributable to an increased relative abundance of Bacteroidetes, consistent with previous dietary studies showing tradeoffs between Firmicutes and Bacteroidetes in obese adults (Ley et al., 2005) and children (Bervoets et al., 2013). Other studies have investigated functional shifts in the salivary microbiome following probiotic administration (Dassi et al., 2014), differences in the pulmonary microbiome of HIV patients in San Francisco vs. Uganda (Iwai et al., 2014), and interplay between human mutation, Crohn’s disease, and the functional repertoire of the gut microbiota (Tong et al., 2014). Finally, HSP methods are also being used to study non-model systems (Loudon et al., 2013; Polónia et al., 2014). For example, significant differences in several gene categories, including tetracycline production, were inferred for two sponge microbiotas vs. nearby seawater or sediment (Polónia et al., 2014).

These examples illustrate how HSP methods are being used in studies that seek to unravel the complex interactions between host factors (genetics, immune function, diet), harmful or helpful microbial symbionts, and downstream functional consequences in diverse organisms.

### FACTORS INFLUENCING THE ACCURACY OF HIDDEN STATE PREDICTION ALGORITHMS

**FIGURE 2:**
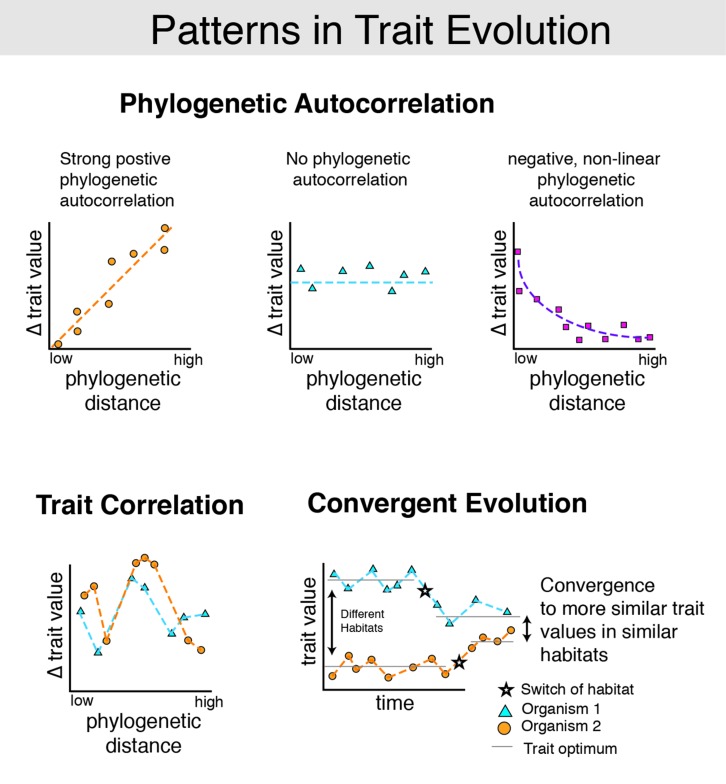
**Common patterns in trait evolution.** Several common, non-mutually exclusive patterns of trait evolution are pictured. For many traits, change over time tends to follow the evolutionary history of the organism. This phenomenon is known as *positive phylogenetic autocorrelation* (top). Phylogenetic autocorrelation has been observed in a wide variety of traits (~60% of 103 traits examined in (Freckleton et al., 2002). For example, closely related mammals have more similar body sizes than distantly related mammals (Gittleman et al., 1996). Phylogenetic autocorrelations are often positive (top left), but may in some cases be negative (top right). For example, closely related organisms inhabiting the same niche may diversify traits to escape competitive exclusion and exploit new resources. This may produce negative phylogenetic autocorrelation in closely related, cohabiting species. In contrast, *trait correlation* occurs when traits are linked to the evolution of other traits. For example, in RNAs where secondary structure is important, some nucleotide positions must match in order to form Watson–Crick base-pairs (e.g., in stems) or be otherwise constrained to preserve pseudoknots (e.g., Savill et al., 2001). Thus if each nucleotide in the gene coding for such an RNA is modeled as a discrete trait, many of these traits will be correlated with one another. Continuous traits may also be correlated with one another (bottom left). Finally, some traits may have undergone *convergent evolution* (bottom right). Examples of convergence are plentiful, ranging from the similar morphology of cacti and euphorbia, which have independently adapted to arid climates, to skull morphology in diverse herbivorous vs. carnivorous lizards (Stayton, 2006). Existing HSP methods work best when traits exhibit strong positive phylogenetic autocorrelation. Statistical methods that account for observed convergent evolution of gene content within habitats (Chaffron et al., 2010; Zaneveld et al., 2010) and negative phylogenetic autocorrelation among co-occurring strains of the same OTU (Zaneveld et al., 2010) remain an important topic for future development.

Hidden State Prediction algorithms will function best when traits exhibit strong, positive phylogenetic autocorrelation (**Figure 2**). Such correlations have been shown for many morphological traits of ecological interest (Freckleton et al., 2002). Comparative genomic studies have also shown phylogenetic autocorrelation for the content of microbial genomes by linking organismal phylogenetic divergence to both the collection of genes in bacterial genomes (Konstantinidis and Tiedje, 2005; Chaffron et al., 2010; Zaneveld et al., 2010; Okuda et al., 2012) and gene order (Tamames, 2001). Although convergent evolution of gene content within habitats (Chaffron et al., 2010; Zaneveld et al., 2010) and local negative phylogenetic autocorrelation have also been described (Zaneveld et al., 2010), these effects are generally of smaller magnitude. Correlation between organismal and functional diversity was also observed by the Human Microbiome Project (HMP), which found richness of gene function to be correlated with the taxonomic richness of microbial consortia (Consortium, 2012).

Because HSP methods predict features of modern organisms, they are readily testable by cross-validation. Tests have included comparison of sequenced vs. predicted genome contents for known genomes; prediction accuracy for synthetic metagenomes constructed *in silico* from sequenced genomes; cross-validation of annotated 16S rRNA copy numbers; and validations on cell and DNA-based mock communities of known composition. Both HSP and related methods (Okuda et al., 2012) have generally reported high accuracy (Kembel et al., 2012; Langille et al., 2013; Angly et al., 2014) with certain important exceptions summarized below.

Specific features that have been shown to compromise the accuracy of HSP methods in particular cases include: (a) low availability of reference data for phylogenetically diverse organisms (Okuda et al., 2012; Langille et al., 2013), (b) lineages that follow an evolutionary process that differs strongly from the evolutionary model used in inference (especially genome reduction in intracellular endosymbionts; Zaneveld et al., 2010; Langille et al., 2013). Other factors that have a more modest (though still statistically significant) effect on accuracy include: (a) differences in classes of gene function thought to correspond to rates of lateral transfer (Langille et al., 2013), (b) local error in the phylogeny (Stone, 2011; Langille et al., 2013); (c) the choice of ASR method (Langille et al., 2013), and (d) substitution of detailed taxonomic trees (with unit length branches) instead of a phylogeny (Angly et al., 2014). Finally, because HSP methods rely on the structure of the phylogenetic tree rather than taxonomy at a particular rank, they are robust to taxonomic labels that in some cases may not adequately reflect ecological strategy (Philippot et al., 2010).

## CONCLUSION

For many traits, phylogeny provides a useful framework for summarizing knowledge gained by studying model taxa. While methods that use phylogeny to predict traits have been available for decades, it is only relatively recently that these methods have been applied at high-throughput to summarize our understanding of key players in microbial symbioses. Several exciting directions are likely to both further improve the accuracy of HSP algorithms in the domain of microbial trait prediction, and open new avenues of research.

The strongest single factor limiting the accuracy of predictions made by HSP is the availability of phylogenetically diverse reference data. In the case of using HSP to predict genome features, the relevant reference data are genome sequences. Genome sequences are used to calculate counts of gene families across microorganisms, which are then used as evolutionary characters in the algorithm. However, the vast majority of genome sequences are incomplete, and therefore cannot be used with existing HSP techniques. For example, as of this writing the PATRIC resource hosts 13,091 partial bacterial genomes vs. 2,544 complete genomes (Wattam et al., 2014). Lack of complete sequencing introduces uncertainty into the counts of gene families in that organism, and thereby complicates use of these sequences as input data for HSP. Statistical tests are needed to determine whether read depth is sufficient to conclude that absence of evidence for a particular gene family in the partial genome sequence represents genuine absence vs. missing data. Further extension of these methods to single cell genomic data (most often incomplete) could potentially allow incorporation of information from many uncultured and understudied phyla (“microbial dark matter”; Rinke et al., 2013). Algorithmic improvements that allow incorporation of information on gene content from partial genome sequences will be an important direction for future HSP algorithms in microbial ecology. For example, Bayesian HSP methods might integrate over distributions of possible copy numbers in partial genomes (derived from analysis of read depth). This is a similar to existing strategies for incorporating uncertainty in the parameters of the evolutionary model or the topology of the phylogenetic tree. Such methods would allow incorporation of much more comprehensive input datasets, and thus will likely represent an important advance in the accuracy of predictive functional profiling with HSP.

In systems where many sequenced genomes are available, HSP might be used to extend metabolic predictions of species interactions (Levy and Borenstein, 2013) where sequence information is lacking. This in turn may help to identify cases in which co-occurrence patterns (i.e., correlated abundance across samples) between two microorganisms may be driven by syntrophic mutualism. Recent advances suggest that this approach will be fruitful. Functional profiles imputed using HSP have been correlated with metabolomics profiles (McHardy et al., 2013), and metabolic modeling applied to test ideas about the processes driving co-occurrence patterns in the human gut microbiome (Levy and Borenstein, 2013). A key test of the utility of HSP methods for this application will be a comparison of the accuracy of metabolic networks built from sequenced genomes vs. the HSP prediction for the genes in that genome. If the loss in accuracy is modest, then HSP could provide a rough outline of potentially interesting metabolic interactions at the level of entire microbial communities that could then be targeted for experimental confirmation.

## Conflict of Interest Statement

The authors declare that the research was conducted in the absence of any commercial or financial relationships that could be construed as a potential conflict of interest.

## Footnotes

1 https://github.com/srjun/PanFP

2 http://picrust.github.io/picrust/
